# The characteristics of asbestos-related disease claims made to the Korea Workers’ Compensation and Welfare Service (KCOMWEL) from 2011 to 2015

**DOI:** 10.1186/s40557-018-0256-6

**Published:** 2018-07-11

**Authors:** Yon Soo An, Hyung Doo Kim, Hyeoung Cheol Kim, Kyoung Sook Jeong, Yeon Soon Ahn

**Affiliations:** 1 Department of Occupational and Environmental Medicine, Soonchunhyang University Cheonan Hospital, 31 Suncheonhyang 6-gil, Dongnam-gu, Cheonan-si, Chungcheongnam-do 31151 Republic of Korea; 2 Department of Occupational and Environmental Medicine, Inha University Hospital, 27 Inhang-ro, Jung-gu, Incheon, 22332 Republic of Korea; 3 Department of Occupational and Environmental Medicine, Kangbuk Samsung Hospital, 29 Saemunan-ro, Jongro-gu, Seoul, 03181 Republic of Korea; 4 Department of Occupational and Environmental Medicine, Hallym University Sacred Heart Hospital, 22 Gwanpyeong-ro 170beon-gil, Dongan-gu, Anyang-si, Gyeonggi-do 14068 Republic of Korea; 5 Department of Preventive Medicine and Institute of Occupational and Environmental Medicine, Wonju College of Medicine, Yonsei University, 20 Ilsan-ro, Wonju, Gangwon-do 26426 Republic of Korea

**Keywords:** Asbestos, Malignant mesothelioma, Lung cancer, Workers’ compensation

## Abstract

**Background:**

This study aimed to enhance understanding of the epidemiologic characteristics of asbestos-related diseases, and to provide information that could inform policy-making aimed at prevention and compensation for occupational asbestos exposure, through analyzing asbestos-related occupational disease claims to Korea Workers’ Compensation and Welfare Service from 2011 to 2015.

**Methods:**

We analyzed 113 workers who filed medical care claims or survivor benefits for asbestos exposure and occupational-related disease from 2011 to 2015. Among these claims, we selected approved workers’ compensation claims relating to malignant mesothelioma and lung cancer, and analyzed the general characteristics, exposure characteristics, pathological characteristics, and occupation and industry distribution.

**Results:**

Malignant mesothelioma and lung cancer occurred predominantly in males at 89.7 and 94%, respectively. The mean age at the time of diagnosis for malignant mesothelioma and lung cancer was 59.5 and 59.7 years, respectively, while the latency period for malignant mesothelioma and lung cancer was 34.1 and 33.1 years, respectively. The companies involving exposed workers were most commonly situated within the Busan-Ulsan-Gyeongnam region. Histology results for lung cancer indicated adenocarcinoma as the most common form, accounting for approximately one half of all claims, followed by squamous cell carcinoma, and small cell lung cancer. The most common occupation type was construction in respect of malignant mesothelioma, and shipbuilding in respect of lung cancer.

**Conclusions:**

Considering the long latency period of asbestos and that the peak period of asbestos use in Korea was throughout the mid-1990s, damage due to asbestos-related diseases is expected to show a continued long-term increase. Few studies providing an epidemiologic analysis of asbestos-related diseases are available; therefore, this study may provide baseline data to assist in predicting and preparing for future harm due to asbestos exposure.

**Trial registration:**

DUIH 2018–02–004-001. Registered 28 Februrary 2018.

## Background

In Korea, extensive exposure to asbestos began with the development of asbestos mines during the 1930s. Asbestos use decreased temporarily from 1945, after liberation from Japanese occupation; however a significant amount of asbestos was imported during the 1970s and was widely used in building materials, machine componentry, and insulation material. Even today, although the use of asbestos in insulation and soundproofing materials has been minimized or banned, workers and citizens are constantly exposed to asbestos during the processes of building demolition and reconstruction [1].

According to Ahn et al. (2009), compensation for occupational malignant mesothelioma and lung cancer associated with asbestos exposure, in accordance with the Industrial Accident Compensation Insurance Act, was made for the first time in 1993 in respect of malignant mesothelioma, and in 1998 for lung cancer. Subsequently, a total 19 cases of malignant mesothelioma and 41 cases of lung cancer were recognized as asbestos-related occupational cancers by 2007 [2]. Moreover, between 1994 and 2011, the percentage of claims involving asbestos exposure among 179 workers who received worker’s compensation for occupational lung cancer was 48.6% (*n* = 87) [3].

However, because there are no official data from the Korea Workers’ Compensation and Welfare Service (KCOMWEL), it is impossible to know the exact scale of worker compensation due to asbestos exposure. Moreover, even the analysis of industrial accidents published annually by the Ministry of Employment and Labor, which are considered to be the only statistics on worker compensation associated with asbestos exposure, only included asbestos-related statistics starting from 2002.

In 2011, the first year when the Ministry of Environment implemented the Asbestos Injury Relief Act, 249 people received compensation for asbestos-related diseases, such as malignant mesothelioma, lung cancer, and asbestosis pulmonum. However, only 22.5% (*n* = 56) of these people had been exposed to asbestos purely from environmental exposure. On the other hand, 25.3% (*n* = 63) were found to have been exposed to asbestos due to their occupation, and 41.4% (*n* = 103) had been exposed to asbestos through both their occupation and environment. The total percentage of people with occupational exposure was 66.7% (*n* = 166) [4]. Therefore, although it is likely that there were many more cases of people contracting asbestos-related diseases than those workers compensated in accordance with the Industrial Accident Compensation Insurance Act, the actual situation could not be identified due to a lack of accurate statistics for such cases.

Internationally, many countries have recognized asbestos as a hazard and have begun implementing laws for restricting the production and use of asbestos. Korea implemented a complete ban on asbestos use, starting in 2009. However, considering the amount and duration of asbestos use in Korea up to that point, a continued increase in asbestos-related damage to health is anticipated [5–7].

Asbestos-related diseases have a long latency period and manifest in various patterns; therefore, management of asbestos exposure may be the most important consideration. This study attempted to analyze asbestos-related diseases that had been listed as occupational disease claims to KCOMWEL between 2011 and 2015. With the findings, the study aimed to improve understanding of the epidemiologic characteristics of these diseases, and to provide useful information to help inform policies for asbestos-exposure prevention and compensation for exposed workers [2].

## Methods

### Data source and claims analysis

We applied to KCOMWEL for access to their database and to epidemiologic survey reports that could identify claims filed for medical treatment or survivor benefits for asbestos exposure-related occupational diseases between 2011 and 2015. We received data for a total of 142 claims. After excluding 10 duplicate claims, 12 claims with information missing in the epidemiologic survey, and 7 claims for diseases unrelated to asbestos exposure (i.e. breast cancer), a total of 113 claims were selected for analysis.

### Analysis details

We classified the asbestos exposure-related diseases into 3 types: malignant mesothelioma, lung cancer, and other respiratory diseases, including asbestosis pulmonum [8–10]. Among these, malignant mesothelioma and lung cancer cases that had been approved as industrial accident claims were analyzed after dividing them into categories based on general characteristics, exposure characteristics, pathological characteristics, and industry and occupation distribution [Table 1].

**Table 1:** Workers’ characteristics concerning approved and unapproved claims. Unit: No. of workers (%)

Variable		Approved	Unapproved
Sex	Male	82 (91.1)	21 (91.3)
	Female	8 (8.9)	2 (8.7)
Age at diagnosis (years)	< 20	0	0
	20–29	0	2 (8.7)
	30–39	2 (2.2)	2 (8.7)
	40–49	7 (7.8)	4 (17.4)
	50–59	38 (42.2)	6 (26.1)
	60–69	26 (28.9)	5 (21.7)
	≥70	17 (18.9)	4 (17.4)
	Mean ± Standard deviation	59.5 ± 8.9 (min: 36, max: 75, median: 58.5)	53.6 ± 14.6 (min: 22, max: 73, median: 58)
Disease name	Malignant mesothelioma	39 (43.3)	6 (26.1)
	Lung cancer	38 (42.2)	15 (65.2)
	Asbestosis pulmonum and other respiratory diseases	13 (14.4)	2 (8.7)
Duration of exposure (years)	< 5	9 (10.0)	7 (30.4)
	5–9	11 (12.2)	4 (17.4)
	10–19	19 (21.1)	5 (21.7)
	20–29	31 (34.4)	6 (26.1)
	30–39	14 (15.6)	1 (4.3)
	≥40	6 (6.7)	0
	Mean ± Standard deviation	20.5 ± 11.6 (min: 1.5, max: 50, median: 20)	11.7 ± 10.5 (min: 0.1, max: 33, median: 8.5)

## Results

### Workers’ characteristics concerning approved and unapproved claims

Among the approved claims, malignant mesothelioma (*n* = 39, 43.3%) and lung cancer (*n* = 38, 42.2%) were most common diseases, followed by asbestosis pulmonum (*n* = 5, 5.6%), and other respiratory diseases (*n* = 8, 8.9%). Other respiratory diseases included interstitial lung disease (n = 3, 3.3%), pleural plaque (*n* = 2, 2.2%), laryngeal cancer (n = 2, 2.2%), and idiopathic pulmonary fibrosis (*n* = 1, 1.1%). Among the unapproved claims, lung cancer (*n* = 15, 65.2%) was the most common disease. The mean duration of exposure for approved and unapproved claims was 20.5 and 11.7 years, respectively, showing a significant difference (Tables 1 and 2).

**Table 2:** Workers’ general characteristics in relation to approved claims. Unit: No. of workers (%)

Variables		Malignant mesothelioma	Lung cancer
Sex	Male	35 (89.7)	36 (94.7)
	Female	4 (10.3)	2 (5.3)
Age at diagnosis (years)	< 20	0	0
	20–29	0	0
	30–39	1 (2.6)	0
	40–49	2 (5.1)	4 (10.5)
	50–59	19 (48.7)	15 (39.5)
	60–69	8 (20.5)	13 (34.2)
	≥70	9 (23.1)	6 (15.8)
	Mean ± Standard deviation	59.5 ± 8.9 (min: 39, max: 75, median: 57)	59.7 ± 8.8 (min: 43, max: 73, median: 59)
Year of diagnosis	1991–1995	0	0
	1996–2000	0	0
	2001–2005	0	2 (5.3)
	2006–2010	8 (20.5)	17 (44.7)
	2011–2015	31 (79.5)	19 (50.0)
Smoking status	Current-smoker	6 (15.4)	13 (34.2)
	Ex-smoker	12 (30.8)	10 (26.3)
	Non-smoker	12 (30.8)	10 (26.3)
	Unknown	9 (23.1)	5 (13.2)
	Mean pack-years ± standard deviation of current and ex-smokers	17.4 ± 14.5 (min: 0.5, max: 50, median: 14.5)	26.3 ± 17.0 (min: 1.5, max: 60, median: 27)
Region	Seoul	6 (15.4)	4 (10.5)
	Incheon/Gyeonggi	12 (30.8)	3 (7.9)
	Daejeon/Chungnam/Chungbuk	1 (2.6)	2 (5.3)
	Gangwon	0	0
	Gwangju/Chonnam/Chonbuk	2 (5.1)	6 (15.8)
	Daegu/Gyeongbuk	1 (2.6)	4 (10.5)
	Busan/Ulsan/Gyeongnam	17 (43.6)	19 (50.0)

### Workers’ general characteristics concerning approved claims

In both malignant mesothelioma and lung cancer, males accounted for the overwhelming percentage: 89.7% (*n* = 35) in malignant mesothelioma and 94.7% (*n* = 36) in lung cancer, respectively. The mean age at diagnosis for malignant mesothelioma and lung cancer was 59.5 and 59.7 years, respectively. Among those approved for malignant mesothelioma, 46.2% (*n* = 18) had a history of smoking, with a mean pack-years of 17.4. Among those approved for lung cancer, 60.5% (*n* = 23) had a history of smoking, with mean pack-years of 26.3. In terms of regions where the workers’ companies were located, the Busan-Ulsan-Gyeongnam region showed the highest percentage for both malignant mesothelioma (*n* = 17, 43.6%) and lung cancer (*n* = 19, 50%) (Tables 2 and 3).

**Table 3:** Workers’ exposure characteristics concerning approved claims. Unit: No. of workers (%)

Variables		Malignant mesothelioma	Lung cancer
Year of first exposure	1950–1959	1 (2.6)	1 (2.6)
	1960–1969	2 (5.1)	5 (13.2)
	1970–1979	24 (61.5)	20 (52.6)
	1980–1989	6 (15.4)	10 (26.3)
	1990–1999	6 (15.4)	2 (5.3)
Age at first exposure (years)	< 20	11 (28.2)	8 (21.1)
	20–29	18 (46.2)	23 (60.5)
	30–39	6 (15.4)	5 (13.2)
	≥40	4 (10.3)	2 (5.3)
	Mean ± Standard deviation	25.6 ± 8.6 (min: 14, max: 47, median: 25)	25.7 ± 7.0 (min: 15, max: 51, median: 25)
Duration of exposure (years)	< 5	6 (15.4)	2 (5.3)
	5–9	4 (10,3)	3 (7.9)
	10–19	6 (15.4)	11 (28.9)
	20–29	14 (35.9)	14 (36.8)
	30–39	8 (20.5)	4 (10.5)
	≥40	1 (2.6)	4 (10.5)
	Mean ± Standard deviation	19.9 ± 11.5 (min: 1.5, max: 42, median: 22)	21.7 ± 11.4 (min: 2.5, max: 50, median: 20)
Latency period (years)	< 5	0	0
	5–9	0	0
	10–19	3 (7.7)	1 (2.6)
	20–29	6 (15.4)	12 (31.6)
	30–39	22 (56.4)	17 (44.7)
	≥40	8 (20.5)	8 (21.1)
	Mean ± Standard deviation	34.1 ± 8.2 (min: 15, max: 54, median: 35)	33.1 ± 8.2 (min: 14, max: 53, median: 32)

### Workers’ exposure characteristics concerning approved claims

The 1970s produced the highest percentage of first exposure to asbestos for malignant mesothelioma (*n* = 25, 61.5%) and for lung cancer (*n* = 20, 52.6%). Concerning age at first exposure to asbestos, the age group between 20 and 29 had the highest percentage of malignant mesothelioma (*n* = 18, 46.2%) and lung cancer (*n* = 23, 60.5%). For malignant mesothelioma and lung cancer, the mean duration of exposure to asbestos was 19.9 and 21.7 years, respectively, while the mean latency period was 34.1 and 33.1 years, respectively (Tables 3 and 4).

**Table 4:** Workers’ pathological characteristics concerning approved claims. Unit: No. of workers (%)

Variables		Malignant mesothelioma	Lung cancer
Primary site (malignant mesothelioma)	Pleura	23 (59.0)	
	Peritoneum	11 (28.2)	
	Mediastinum	2 (5.1)	
	Unknown	3 (7.7)	
Primary site (lung cancer)	Lower lobe		18 (47.4)
	Upper lobe or middle lobe		16 (42.1)
	Mediastinum		1 (2.6)
	Hilum		1 (2.6)
	Unknown		2 (5.3)
Pathologic findings (lung cancer)	Non-small cell lung cancer		33 (86.8)
	Adenocarcinoma		19 (50.0)
	Small cell carcinoma		12 (31.6)
	Large cell carcinoma		0
	Bronchioalveolar carcinoma		0
	Unknown		2 (5.3)
	Small cell lung cancer		3 (7.9)
	Carcinoid		0
	Unknown		2 (5.3)

### Workers’ pathological characteristics concerning approved claims

The most common primary site of malignant mesothelioma was the pleura (n = 23, 59.0%), followed by the peritoneum (*n* = 11, 28.2%). Among lung cancer claims, a higher percentage of cancers were located in the lower lobe (*n* = 18, 47.4%) than in the upper and middle lobes combined (*n* = 16, 42.1%). Histological analysis of lung cancer claims showed that 19 claims involved adenocarcinoma, which accounted for half of all claims, followed by squamous cell carcinoma (*n* = 12, 31.6%) and small cell lung cancer (*n* = 3, 7.9%) (Tables 4 and 5).

**Table 5:** Workers’ distribution by industry and occupation concerning approved claims. Unit: No. of workers

Malignant mesothelioma		Type of exposure	Lung cancer	
Industrial classification	Job classification		Industrial classification	Job classification
Manufacture of asbestos textiles (4)	Manufacturer of textile containing asbestos (4)	Manufacture of products using asbestos as raw material	Manufacture of slates (5)	Manufacturer of slate (5)
Manufacture of electronic components (3)	Manufacturer of commutator containing asbestos (3)		Manufacture of parts and accessories for motor vehicles (2)	Manufacturer of brake lining (2)
Manufacture of slates (1)	Manufacturer of slate (1)		Manufacture of asbestos textiles (2)	Manufacturer of textile containing asbestos (2)
Manufacture of stone products (1)	Manufacturer of firebrick containing dolomite (1)		Manufacture of asbestos boards (2)	Manufacturer of asbestos board (2)
Manufacture of pipes and tubes (1)	Worker applying asbestos to pipe for water supply (1)	Use of asbestos-containing products in manufacturing process	Manufacture of boilers (1)	Manufacturer of boiler using asbestos as insulating material (1)
Construction (12)	Plumber (5)	Handling asbestos-containing products during work	Building of ships (8)	Welder (6)
	Slate roof painter (1)			
				Cleaner of waste in ship (1)
	Building maintenance and repairman (1)			
				Ship demolition worker (1)
			Construction (4)	Plumber (2)
	Interior product installer (1)			
				Duct installer (1)
	Worker repairing roof (1)			
				Building demolition worker (1)
			Repair services of motor vehicles (3)	Maintenance and repairman (3)
	Duct installer (1)			
	Building construction supervisor (1)			
	Building demolition worker (1)			
			Petroleum refineries (2)	Boiler operator (2)
Construction of installing electric equipment (4)	Electric equipment installation and repairman (2)			
			Manufacture of basic iron and steel (1)	Cleaner of warehouse containing asbestos products (1)
	Electricity and cable setting man (1)			
	Elevator installer (1)			
			Construction: installing electric equipment (1)	Closed-circuit television installer (1)
Building of ships (3)	Welder (1)			
	Plumber (1)			
	Ship demolition worker (1)			
			Cleaning services (1)	Cleaner of demolished building (1)
Manufacture of basic iron and steel (2)	Smelting furnace worker (2)			
Manufacture of motor vehicles (1)	Car assembler (1)			
			Defense activities (1)	Electricity and cable setting (1)
Manufacture of parts and accessories for motor vehicles (1)	Warehouse keeper (1)			
			Transport via railways (1)	Maintenance and repairman (1)
Repair services of motor vehicles (1)	Maintenance and repairman (1)			
Transport via railways (1)	Railroad construction supervisor (1)			
			Medical services (1)	Boiler operator (1)
Manufacture of rail locomotives and rolling stock (1)	Welder (1)			
Cleaning services (1)	Street cleaner (1)			
			Thermal power generation (1)	Plumber (1)
Manufacture of textiles (1)	Boiler operator (1)			
Cargo handling (1)	Cargo worker (1)			
		Miscellaneous	Mining (2)	Asbestos mine worker (1)
				Asbestos mine investigator (1)

### Workers’ distribution by industry and occupation concerning approved claims

Industry types were classified into 4 categories based on existing relevant studies: 1) manufacture of products using asbestos as a raw material; 2) use of asbestos-containing products in the manufacturing process; 3) handling of asbestos-containing products during work; and 4) miscellaneous [11]. Each industry type was subdivided according to the 10th edition of the Korean Standard Industrial Classification (KSIC).

The highest number of malignant mesothelioma cases involved those working in industries that handled asbestos-containing products (*n* = 29), followed by industries that manufactured products using asbestos as a raw material (*n* = 9), and using asbestos-containing products in the manufacturing process (*n* = 1). The highest number of claims were identified within the construction industry (*n* = 12), followed by the manufacturers of asbestos textiles (*n* = 4), and those installing electrical equipment (n = 4) (Table 5).

The highest number of lung cancer cases was found in industries involved in handling asbestos-containing products at work (*n* = 24), followed by industries involved in manufacturing products using asbestos as a raw material (*n* = 11), miscellaneous (n = 2), and industries involved in the use of asbestos-containing products within the manufacturing process (n = 1). The highest number of lung cancer cases were found in the shipbuilding industry (*n* = 8), followed by the slate manufacturing industry (*n* = 5) (Table 5).

## Discussion

Among the diseases for which claims were approved, asbestosis pulmonum (n = 5, 5.6%) occurred less frequently than malignant mesothelioma and lung cancer. In the past, detailed criteria for determining asbestosis pulmonum in the enforcement decree of the Industrial Accident Compensation Insurance Act have been insufficient. However, with the implementation of asbestos screening meetings in 2016, the criteria for disability and treatment related to asbestosis pulmonum became more specific, which may result in a change to the percentage of asbestosis pulmonum cases in future.

In 1987, the International Agency for Research on Cancer (IARC) classified asbestos as a Group 1 carcinogen capable of causing malignant mesothelioma, lung cancer, asbestosis pulmonum, pleural disease, laryngeal cancer, and ovarian cancer in humans [12]. We identified 2 workers who received approval for laryngeal cancer claims recognized as industrial accidents. One worker employed for 13 years at a shipyard was exposed to asbestos as a result of installing ceilings, walls, and partitions inside ship cabins, while another worker was exposed to asbestos at construction sites while employed for 38 years as a supervisor for a construction company.

The present study identified 39 cases of malignant mesothelioma and 38 cases of lung cancer associated with asbestos exposure which were approved for industrial accident compensation during the 5-year period from 2011 to 2015. These figures represented a significant increase from 19 and 41 approved cases of malignant mesothelioma and lung cancer, respectively, over a 15-year period between 1993 and 2007 [2].

Meanwhile, 3664 people filed for asbestos injury relief during a 7-year period between 2011 and 2017, which far exceeded the number of those who filed for industrial accident insurance and, among them, 2599 received approval. In light of a 2012 report by the Korea Environment Corporation, it is suspected that approximately two-thirds of the people who received approval may also be those who may have had occupational asbestos exposure [4]. Considering this, the actual number of people with asbestos-related diseases due to occupational exposure may be much higher than reported in the present study. However, because there are no accurate statistics on this, the current status cannot be determined [2].

Statistics on malignant mesothelioma in Korea reported a sex ratio of 195.6; however, the sex ratio in the present study was 875.0 [13]. We consider the reason for this large ratio difference is due to asbestos-exposure occupations tending to involve a relatively higher percentage of male workers.

In previous studies, the mean age at diagnosis with malignant mesothelioma and lung cancer was 53.1 and 50.6 years, respectively [2], whereas the mean age in the present study was higher at 59.5 and 59.7 years, respectively. Such differences may be attributed to the large volume of imported asbestos used in building materials, machine componentry, and insulation materials during economic development in the 1970s, coupled with the latency period of asbestos-related diseases being between 10 and 50 years [1, 14]. Meanwhile, the mean latency period of malignant mesothelioma and lung cancer in previous studies has been reported as 22.6 and 22.1 years, respectively [2], whereas the mean latency period in our study was much longer, at 34.1 and 33.1 years, respectively.

Gemba et al. (2012) reported that the age of 607 patients at the time of malignant mesothelioma diagnosis associated with occupational exposure to asbestos in Japan was between 25 and 94 years (median, 68 years), while the latency period was between 13 and 81 years (median, 43 years) [15]. Kishimoto et al. (2003) reported that the age of 120 patients at the time of diagnosis with asbestos-related lung cancer was between 47 and 87 years (median, 70 years), while the latency period was between 15 and 69 years (median, 43 years) [16]. These two Japanese studies reported higher ages at diagnosis and longer latency periods than results from Korean studies.

The highest percentage of workers approved for lung cancer due to asbestos exposure have been reported as coming from the Busan, Ulsan, and Gyeongnam region at 29.2% [2]; however, our study identified 50% of workers coming from the same region. We consider this higher percentage was due to the first asbestos textile factory being established in 1969 in Busan, and subsequently, most of the factories that manufactured asbestos textiles being located in the Busan, Ulsan, and Gyeongnam region, while ship repair and shipbuilding industries began to be fully operational in the same region from the 1970s [5, 14]. The Kang et al. (2016) study supports this interpretation in their analysis of changes in the distribution of asbestos-related industries in Korea using different timeframes [17].

From a histological lung cancer perspective, a previous study reported in descending order adenocarcinoma (62.5%), followed by small cell lung cancer (18.8%), and squamous cell carcinoma (15.6%) [2], whereas the present study identified a slight difference in order with adenocarcinoma (50.0%), squamous cell carcinoma (31.6%), and small cell lung cancer (7.9%). According to a study by Kishimoto et al. (2010), the histological classification of 152 Japanese patients with asbestos-related lung cancer showed the same order as the present study with adenocarcinoma (55.9%), squamous cell carcinoma (25.7%), and small cell lung cancer (11.8%) [18]. Moreover, a study by Uguen et al. (2017) on the histological distribution of 146 French patients with asbestos exposure-related lung cancer also reported the same order as the present study with adenocarcinoma (45.9%), squamous cell carcinoma (38.4%), and small cell lung cancer (4.8%) [19]. Meanwhile, according to data published in 2016 by the Korea Central Cancer Registry, histological classification of all 24,007 cases of lung cancer that occurred in Korea in 2014 also showed the same order of adenocarcinoma (43.5%), squamous cell carcinoma (22.5%), and small cell lung cancer (11.4%) [20]. In summarizing these results, asbestos-related lung cancer in Korea seems to follow the same histological distribution of asbestos-related lung cancer in other countries and general lung cancer among Koreans.

Under the current laws, government compensation for people who have contracted an asbestos-related disease is either assessed through the Industrial Accident Compensation Insurance Act that compensates claims involving occupational exposure, or through the Asbestos Injury Relief Act that compensates claims involving environmental exposure. However, asbestos has a very long latency period until it causes an associated disease, and many businesses that handle asbestos are small and most of those businesses have closed due to decreased asbestos use. Therefore, victims of previous occupational exposure to asbestos face difficulties proving that they actually performed asbestos-related work.

Compensation for victims of occupational asbestos exposure through industrial accident insurance, and not through the Asbestos Injury Relief Act, is more in line with the original intent of the Industrial Accident Compensation Insurance Act. Moreover, from an economic perspective, compensation paid through industrial accident insurance is much higher than that paid through the Asbestos Injury Relief Act. Therefore, for the occupational asbestos exposure victims who have difficulty proving their work history, it is necessary to recognize a variety of data sources, such as the testimony of co-workers, company personnel data, industrial accident insurance, health insurance, and employment insurance, in a similar manner to that of patients with coniosis who worked in the mining industry. If the current system of issuing health management pocketbooks could be reinforced to identify the source of exposure and high-risk groups, such measures may help to predict and manage future occurrences of asbestos-related diseases [14]. Moreover, instead of adopting a passive stance in simply accepting that compensatory relief will be available for the future onset of asbestos-related disease, more attention should be paid to asbestos-related disease prevention projects, including highlighting the dangers of asbestos and providing further education concerning the risks of smoking in asbestos exposure-related high-risk groups.

The present study could not analyze the data of those who had a history of asbestos-related work among those approved for compensation under the Asbestos Injury Relief Act between 2011 and 2017. Therefore, we were not able to accurately identify disease due to occupational exposure to asbestos and this was a limitation of the study. However, the study analyzed all workers who were approved for compensation under the Industrial Accident Compensation Insurance Act for asbestos-related diseases in the 5-year period between 2011 and 2015. The findings of the present study may be useful in establishing new policies and provide improved understanding of asbestos-related diseases among Korean workers.

## Conclusions

The latency period of asbestos is known to be between 10 and 50 years, while there has been a gap of between 30 and 40 years from when each country began regulating asbestos use to incidences of related diseases beginning to decrease [14, 21]. Accordingly, considering that asbestos has a long latency period and the peak period of asbestos use in Korea was the mid-1990s, the damage caused by asbestos-related diseases is expected to show a continued increasing long-term trend. According to Kwak et al. (2017), 3579 new cases of malignant mesothelioma are anticipated in Korea over the next 20 years, and the incidence rate is also expected to be higher between 2029 and 2033 than between 2009 and 2013 [22].

Given there have been few studies on epidemiologic analysis on asbestos-related diseases, the findings in the present study provide baseline data for predicting and preparing for future occurrences of asbestos-related disease. In addition, these findings may play a key role in promoting understanding of the characteristics of asbestos-related occupational diseases, and may guide policies designed to prevent and compensate for occupational exposure to asbestos [2].

## References

[1] Kim HR, Ahn Y-S, Jung S-H (2009). Epidemiologic characteristics of malignant mesothelioma in Korea. J Korean Med Assoc.

[2] Ahn YS, Kang SK (2009). Asbestos-related occupational cancers compensated under the industrial accident compensation Insurance in Korea. Ind Health.

[3] Ahn Y-S, Jeong KS (2014). Epidemiologic characteristics of compensated occupational lung cancers among Korean workers. J Korean Med Sci.

[4] Corporation KE: Performance report of asbestos injury relief act in Korea in 2011 In.: Ministry of Environment; 2012.

[5] Koo J-W, Kim HR (2009). Occupational and environmental asbestos exposure in Korea. J Korean Med Assoc.

[6] LaDou J, Castleman B, Frank A, Gochfeld M, Greenberg M, Huff J, Joshi TK, Landrigan PJ, Lemen R, Myers J (2010). The case for a global ban on asbestos. Environ Health Perspect.

[7] Rosner D, Markowitz G (2017). "Ain't necessarily so!": the brake Industry's impact on asbestos regulation in the 1970s. Am J Public Health.

[8] Jamrozik E, de Klerk N, Musk AW (2011). Asbestos-related disease. Intern Med J.

[9] O'Reilly KM, McLaughlin AM, Beckett WS, Sime PJ (2007). Asbestos-related lung disease. Am Fam Physician.

[10] Prazakova S, Thomas PS, Sandrini A, Yates DH (2014). Asbestos and the lung in the 21st century: an update. Clin Respir J.

[11] Choi SJ, Paek NW, Seok MH, Park JI, Lee SY, Lee YK: A Study on the prevention of adverse health effects caused by asbestos. In*.*: Occupational Safety and Healty Research Institute; 2006.

[12] Marsili D, Terracini B, Santana VS, Ramos-Bonilla JP, Pasetto R, Mazzeo A, Loomis D, Comba P, Algranti E (2016). Prevention of asbestos-related disease in countries currently using asbestos. Int J Environ Res Public Health.

[13] Jung SH, Kim HR, Koh SB, Yong SJ, Chung MJ, Lee CH, Han J, Eom MS, Oh SS (2012). A decade of malignant mesothelioma surveillance in Korea. Am J Ind Med.

[14] Kang D-M, Gu D-C, Kim K-H (2009). Asbestos-related diseases among asbestos textile factory workers and residents around the factory. J Korean Med Assoc.

[15] Gemba K, Fujimoto N, Kato K, Aoe K, Takeshima Y, Inai K, Kishimoto T (2012). National survey of malignant mesothelioma and asbestos exposure in Japan. Cancer Sci.

[16] Kishimoto T, Ohnishi K, Saito Y (2003). Clinical study of asbestos-related lung cancer. Ind Health.

[17] Kang D-M, Kim J-E, Kim J-Y, Lee H-H, Hwang Y-S, Kim Y-K, Lee Y-J (2016). Environmental asbestos exposure sources in Korea. Int J Occup Environ Health.

[18] Kishimoto T, Gemba K, Fujimoto N, Onishi K, Usami I, Mizuhashi K, Kimura K (2010). Clinical study of asbestos-related lung cancer in Japan with special reference to occupational history. Cancer Sci.

[19] Uguen M, Dewitte JD, Marcorelles P, Loddé B, Pougnet R, Saliou P, De Braekeleer M, Uguen A (2017). Asbestos-related lung cancers: a retrospective clinical and pathological study. Mol Clin Oncol.

[20] Registry KCC: Annual report of cancer statistics in Korea in 2014. In*.*: Ministry of Health and Welfare; 2016.

[21] Lin RT, Takahashi K, Karjalainen A, Hoshuyama T, Wilson D, Kameda T, Chan CC, Wen CP, Furuya S, Higashi T (2007). Ecological association between asbestos-related diseases and historical asbestos consumption: an international analysis. Lancet.

[22] Kwak KM, Paek D, Hwang SS, Ju YS (2017). Estimated future incidence of malignant mesothelioma in South Korea: projection from 2014 to 2033. PLoS One.

